# Cost-effectiveness analysis of colorectal cancer screening with stool DNA testing in intermediate-incidence countries

**DOI:** 10.1186/1471-2407-6-136

**Published:** 2006-05-24

**Authors:** Grace Hui-Min Wu, Yi-Ming Wang, Amy Ming-Fang Yen, Jau-Min Wong, Hsin-Chih Lai, Jane Warwick, Tony Hsiu-Hsi Chen

**Affiliations:** 1 Graduate Institute of Epidemiology, College of Public Health, National Taiwan University, Taipei, Taiwan; 2 Institute of Preventive Medicine, College of Public Health, National Taiwan University, Taipei, Taiwan; 3 Department of Internal Medicine, National Taiwan University Hospital, Taipei, Taiwan; 4 School of Medical Technology, College of Medicine, National Taiwan University, Taipei, Taiwan; 5 Cancer Research UK Department of Epidemiology, Mathematics and Statistics, Wolfson Institute of Preventive Medicine, Queen Mary University of London, UK; 6 Division of Biostatistics, Graduate Institute of Epidemiology, College of Public Health, National Taiwan University, Taiwan

## Abstract

**Background:**

The aim of this study is to compare the cost-effectiveness of screening with stool DNA testing with that of screening with other tools (annual fecal occult blood testing, flexible sigmoidoscopy every 5 years, and colonoscopy every 10 years) or not screening at all.

**Methods:**

We developed a Markov model to evaluate the above screening strategies in the general population 50 to 75 years of age in Taiwan. Sensitivity analyses were performed to assess the influence of various parameters on the cost-effectiveness of screening. A third-party payer perspective was adopted and the cost of $13,000 per life-year saved (which is roughly the per capita GNP of Taiwan in 2003) was chosen as the ceiling ratio for assessing whether the program is cost-effective.

**Results:**

Stool DNA testing every three, five, and ten years can reduce colorectal cancer mortality by 22%, 15%, and 9%, respectively. The associated incremental costs were $9,794, $9,335, and $7,717, per life-year saved when compared with no screening. Stool DNA testing strategies were the least cost-effective with the cost per stool DNA test, referral rate with diagnostic colonoscopy, prevalence of large adenoma, and discount rate being the most influential parameters.

**Conclusion:**

In countries with a low or intermediate incidence of colorectal cancer, stool DNA testing is less cost-effective than the other currently recommended strategies for population-based screening, particularly targeting at asymptomatic subjects.

## Background

Because of the high incidence, long preclinical period, and availability of treatment which gives a favorable prognosis with early diagnosis, screening for colorectal cancer (CRC) has been shown to lead to substantial mortality reductions in Western countries; 15–33% with fecal occult blood testing (FOBT), 33% with flexible sigmoidoscopy and 57% with colonoscopy [1-7]. The U.S. Multisociety Task Force on Colorectal Cancer has therefore recommended multiple options for screening people at average risk of CRC including annual FOBT, flexible sigmoidoscopy every 5 years, and colonoscopy every 10 years [8].

Given this choice of effective screening tools, the deciding factor amongst alternatives could be efficacy, performance (sensitivity and specificity), acceptability, feasibility, compliance, and clinical capacity. Colonoscopy, for example, has been recommended as one of the screening options in the USA because of high sensitivity and specificity [8]. However, in countries with a shortage of medical endoscopic manpower the resulting increased demand for colonoscopy may render such a scheme unworkable. In addition, the uptake of colonoscopy is likely to depend upon local social and cultural issues.

Recently, a new method for detecting adenoma and invasive CRC, known as stool DNA testing, has been suggested as a potential screening tool [8,9]. It analyzes the DNA contained in stools, through natural exfoliation, and detects alterations. The sensitivity of stool DNA testing, based on symptomatic cases, has been reported as between 36% and 82% for advanced adenoma and between 61% and 100% for invasive cancer, and the specificity has been estimated at between 89% and 100% in clinical studies [10-15]. A recent large prospective study, targeting average-risk, asymptomatic subjects aged 50 years or older, reported more conservative results when using stool DNA testing as a screening tool; sensitivities of 15% and 52% respectively for adenoma and invasive cancer [16]. Nevertheless, stool DNA testing was found significantly better than the fecal occult blood test [16]. Since the costs associated with stool DNA testing are considerable the issue of whether the required expenditure could be offset by future savings (brought about by a reduction in the number of advanced cases needing treatment) must be addressed before stool DNA testing, as a population-based screening tool for average-risk, asymptomatic subjects, can be introduced. This is particularly important for countries with a low or intermediate, but nevertheless dramatically increasing, incidence of CRC.

In Taiwan for instance, CRC has been ranked as the fourth most common cancer and accounted for 11% of cancer cases and 12% of cancer deaths in 2000 [17]. The age-adjusted incidence has increased by 50%, from 19.4 per 100,000 in 1995 to 28.3 per 100,000 in 2000, while the associated mortality has increased from 11.3 to 12.7 per 100,000.

The aim of this study is therefore to perform a decision analysis using a Markov model to compare the effectiveness and cost of stool DNA testing with other conventional screening strategies. The cost-effectiveness analysis compares triennial, five-yearly, and ten-yearly stool DNA testing (DNA_3_, DNA_5_, and DNA_10_), with no screening (No Screening), annual FOBT (FOBT_1_), five-yearly flexible sigmoidoscopy (SIGM_5_), and ten-yearly colonoscopy (COLO_10_).

## Methods

### Model specification

We developed a Markov model by using DATA Version 3.5 (TreeAge Software) to consider several screening strategies for CRC, including DNA_3_, DNA_5_, DNA_10_, FOBT_1_, SIGM_5_, COLO_10_, and No Screening. Subjects at average-risk of developing CRC were screened from age 50 years until age 75 or death. For each Markov decision, all possible transition states radiated from the decision node with 1-year Markov cycles. Effectiveness was defined as additional life-years gained as a result of screening.

**Figure 1:**
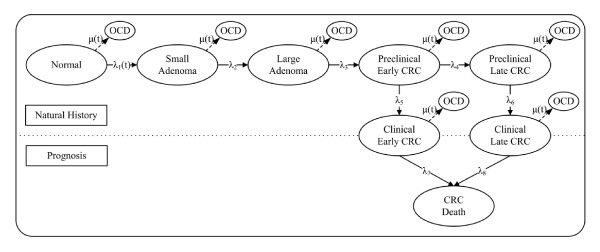
Markov process for disease natural history and prognosis of colorectal cancer (CRC).* * The ovals represent Markov states; the solid arrows represent the direct transition between states, and the dotted arrows represent the transition toward competing cause of death. Abbreviations: Small adenoma, adenoma smaller than 1 cm in size; large adenoma, adenoma larger than 1 cm in size; early CRC, Dukes' stage A and B colorectal cancer, late CRC, Dukes' stage C and D colorectal cancer; OCD, other cause of death.

The disease natural history of CRC was simulated by a nine-state Markov model (Figure 1) consisting of normal, small adenoma (adenoma smaller than 1 cm in size), large adenoma (adenoma larger than 1 cm in size), preclinical early CRC (preclinical Dukes' stage A and B CRC), preclinical late CRC (preclinical Dukes' stage C and D), clinical early CRC, clinical late CRC, CRC death, and other cause of death. Our model only focuses on modeling adenoma-carcinoma sequences without considering non-polypoid CRC because information on the sensitivity and specificity of the screening tool for detecting the non-polypoid form is unclear. The progression from each state to the next follows a Markov process that assumes that transition depends only on the current state, i.e. the process has no memory. The solid arrows represent the direct transition between states, and the dotted ones the transition toward other cause of death. The transition rates are denoted as *λ*_1_(t), *λ*_2_, ..., *λ*_8_, and *μ*(t) where, for example, *λ*_2 _is the annual transition rate from small to large adenoma, and *μ*(t) is the annual age-specific mortality rate from other causes. Note that as the annual incidence rate of small adenoma increases with age we allow it to vary with time according to the Weibull distribution denoted as *λ*_1_(t), i.e. annual incidence rate of small adenoma in subjects aged t. The hazard function for the Weibull distribution is

*λ*_1_(t) = *λ*_10_*γt*^*γ*-1^

where *λ*_10 _and *γ *are the scale and shape parameters respectively. We assume the other transition parameters, *λ*_2_-*λ*_8_, are constant over time. The estimated transition rates are shown in Table 1.

Following Cox and Miller [18], Duffy et al [19] and Chen et al [20], the corresponding annual transition probabilities from one state to another can be obtained by converting the transition rates. The Markov model specifying the transitions between states in Figure 1 illustrates the progressive property of model. At any instant a one-step transition from small adenoma to clinical CRC is not allowed but a multi-step transition is possible, although the likelihood of transition from small to large adenoma, and from large adenoma to pre-clinical CRC, within a year starts low but increases with time. To build this feature into our model, we allowed multiple-step transitions even within one cycle to be possible although the chance is low. Thus, although small adenoma has only a slim chance of progressing through large adenoma, preclinical early CRC, and then to clinical early CRC chronologically within one cycle but our model nevertheless allows for the possibility. Furthermore, since we wanted to simulate a screening scenario, the initial state may be normal, adenoma, or preclinical CRC with the corresponding prevalences at age 50 years. However, there are still lines emanating from the root Markov node to clinical CRC, CRC death, and other death, but the probabilities of these states at the first cycle (initial probabilities) are all zero.

#### Description of screening strategies

**Figure 2:**
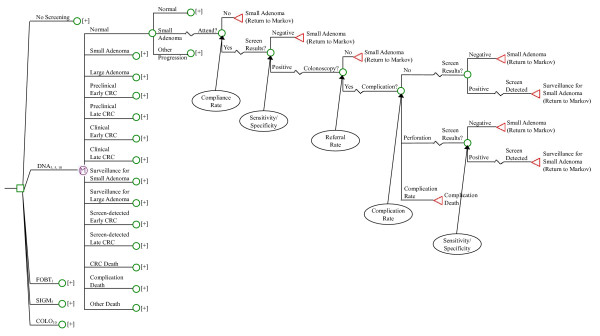
Structure of decision tree model.* * At the beginning of the first Markov cycle, all the probabilities of being in clinical early CRC, clinical late CRC, surveillance for small adenoma, surveillance for large adenoma, screen-detected early CRC, screen-detected late CRC, CRC death, complication death, and other death are zero. Ovals are the chance nodes governed by the probability related to compliance rate, sensitivity, specificity, and referral rate.: Markov cycle. Abbreviations: CRC, colorectal cancer; DNA_3_, stool DNA testing every 3 years; DNA_5_, stool DNA testing every 5 years; DNA_10_, stool DNA testing every 10 years; FOBT_1_, fecal occult blood testing every year; SIGM_5_, sigmoidoscopy every 5 years; COLO_10_, colonoscopy every 10 years; Small adenoma, adenoma smaller than 1 cm in size; large adenoma, adenoma larger than 1 cm in size; early CRC, Dukes' stage A and B colorectal cancer; late CRC, Dukes' stage C and D colorectal cancer.

The "No Screening" strategy follows the disease natural history. The screening procedures for other screening strategies are shown in Figure 2. In stool DNA screening strategies, subjects in the normal, adenoma, or preclinical phase of CRC states will be offered screening but may, or may not, take up stool DNA testing. After modeling the uptake of stool DNA testing, the number of detected cases of adenoma or preclinical CRC is determined by the sensitivity and specificity. A proportion of subjects with positive stool DNA test results will undergo further examination (colonoscopy to detect adenoma and preclinical CRC) as fixed by the compliance rate. The complications of perforation and death due to colonoscopy are also taken into account. Subjects who do not participate in stool DNA testing, those with false negative results and those who refuse diagnostic colonoscopy will return to the disease natural history. Subjects detected with adenoma follow the surveillance procedure and screen-detected cases of CRC follow the prognosis of CRC. The Markov decision model for FOBT screening and sigmoidoscopy screening is similar to that for screening by stool DNA testing, but with different values for sensitivity and specificity. With colonoscopy screening, the procedure for positive results or the surveillance of adenoma is also similar to that for stool DNA testing screening, except that polyps detected with colonoscopy will be removed directly.

Adenoma detected by screening will be removed by polypectomy during first colonoscopy. In the light of the American Cancer Society guidelines on screening and surveillance for the early detection of colorectal adenomas and cancer, the removal of adenoma will be followed up with surveillance [9]. Any recurrence of neoplasm will follow the disease natural history. Subjects with small adenoma will receive colonoscopy after 5 years. Then, if normal, they return to the normal state in the natural history and are screened according to the standard screening strategies. A similar procedure is applied to subjects found to have a large adenoma. They will receive colonoscopy three years after the initial polypectomy and, assuming no adenoma after two repeated colonoscopies, return to the normal state in the natural history mode.

### Base-case estimates

Base-case estimates of the disease natural history and prognosis, screening and diagnostic test characteristics, and costs for cost-effectiveness analysis were abstracted from published literature and are listed in Table 1. Note that the base-case estimates of sensitivity and specificity of stool DNA testing were obtained from the Imperiale et al [16] study that has been so far the only one considering average risk asymptomatic subjects aged 50 years.

#### Transition parameters

Parameters for the disease natural history and prognosis were derived from previous studies [2-4,17,22-24]. The transition rates between various invasive carcinoma states, *λ*_4_-*λ*_6_, were obtained from selective screening for CRC, the Taiwan Multicenter Cancer Screening (TAMCAS) Project [21]. Regarding prevalence rate of adenoma, the prevalence estimates for large adenoma in previous randomised trials [2-4] and the cumulative risk of 35% at 20 years of carcinoma developing from large adenoma [21] coupled with the age-specific CRC incidence rates in Taiwan in 2000 [17], were used to project the age-specific preclinical incidence of small adenoma, *λ*_1_(t) (assuming a Weibull distribution), the transition rate from small to large adenoma, *λ*_2_, (assuming an exponential distribution) and the transition rate from large adenoma to preclinical early CRC [22], *λ*_3 _(assuming an exponential distribution). The estimated age-specific preclinical incidences of small adenoma are shown in Table 1. Assuming survival time follows exponential distribution, the parameters for survival with clinical CRC, *λ*_7 _and *λ*_8_, were derived from the survival probabilities for each stage of CRC [23] after weighting the distribution of CRC stage among the control groups according to previous studies [2-4]. Age-specific mortality, *μ*(t), for other cause of death, refer to Taiwan's vital statistics in 2002 [24]. The prevalence of colorectal neoplasm at age 50 years was obtained by converting the transition rates mentioned above and are shown in Table 1.

#### Test characteristics

The estimated parameters for test characteristics such as sensitivity, specificity, and complication with colonoscopy were derived from previous studies [2-4], [10-14,16,25-40]. This sensitivity is allowed to vary with state. The base-case estimates of sensitivity of stool DNA testing were 8% for small adenoma, 15% for large adenoma and 52% for invasive cancer [16]. The base-case estimate of specificity of stool DNA testing was 94% [16].

#### Attendance and compliance

In the light of reality from previous study, as compliance rates and referral rate vary with screening tools, 60% of compliance rate was assumed for FOBT referring to evidence from several randomised trials [1-4], and 40% of compliance rate was assumed for sigmoidoscopy and colonoscopy in the light of reality from previous studies [41,42]. The compliance with stool DNA testing was assumed to be the same with FOBT. The referral rate to diagnostic colonoscopy after positive findings with screening was assumed to be 85% according to several randomised trials [1-4].

#### Costs

Costs are expressed in US dollars (1 US dollar = 34 New Taiwanese dolloar, exchange rate on the basis of the year 2004). As the cost-effectiveness analysis is carried out from the third-party payer perspective only direct costs were calculated in this study, including those relating to the screening itself, treatment and diagnostic tests (adenoma and CRC only), complications from colonoscopy and surveillance (adenoma only). All estimates of cost refer to the price mandated by the Bureau of National Health Insurance in 2004 or expert opinion. Screening costs, except for stool DNA testing, are based on Medicare Payments by the Bureau of National Health Insurance. The cost of stool DNA testing was estimated by experts after considering the cost of the required laboratory manpower and relevant materials in Taiwan. Treatment and confirmation costs, which relate to polypectomy, biopsy, and pathological examination, were acquired from Medicare Payments by the Bureau of National Health Insurance. The lifetime costs for CRC encompass the initial costs regarding surgery, chemotherapy and radiotherapy, the continuing costs of follow-up after potentially curative therapy, and the eventual cost of terminal care. For the purposes of this costing, expenditure on terminal care is only included for those who (in our models) die from CRC. For early CRC, the initial cost includes only surgery. All future costs and life-years were discounted to the present value at an annual rate of 3%.

### Cost-effectiveness analysis

#### Incremental cost-effectiveness ratio

The comparisons between the "No Screening" and other screening strategies were first evaluated on the basis of the incremental cost-effectiveness ratio (ICER), which is defined as the difference between the two screening groups in terms of cost divided by the difference between them in terms of effectiveness, i.e. the extra cost required to save additional life-years. The ICERs for stool DNA testing as compared to the other screening strategies were also calculated. A cost of $13,000 per life-year saved, which is approximately equivalent to per capita GNP in Taiwan in 2003, was chosen as the ceiling ratio for assessing whether the program is cost-effective.

#### Sensitivity analysis

As several parameters are uncertain, including the prevalence of colorectal neoplasm at age 50 years, transition rates, sensitivity and specificity of screening tool, cost of per unit of stool DNA testing, compliance to screening tool, referral rate to diagnostic colonoscopy, and cost of treatment, a series of one-way sensitivity analyses were performed to assess the influence of changing these parameters on the ICER results. The ranges of variables used in sensitivity analysis are shown in Table 1. Note that improved or worsened sensitivity for small adenoma may lead to changes in sensitivity further down the line, for example, with regard to large adenoma. In this study we assume that any changes in sensitivity to detect small adenoma are paralleled by an equivalent change in sensitivity to detect large adenoma. Similar assumptions were made regarding early and late invasive cancer.

Furthermore, as the previous studies have rather dissimilar estimates for sensitivity and specificity of stool DNA testing, sensitivity analyses were emphasized on the demonstration of the influence of sensitivity and specificity regarding stool DNA testing using the moderate case scenario in which the sensitivities and specificity were obtained from the meta-analysis based on previous studies [10-13,16], and the best case scenario, in which the sensitivities were mainly based on the estimates from Ahlquist study [10] except that the sensitivity of small adenoma was also based on Imperiale study [16]. The sensitivity of stool DNA testing for small adenoma, large adenoma, and colorectal cancer, and the specificity are 8%, 18%, 85%, and 94% in the moderate case scenario. The corresponding estimates in the best case scenario are 8%, 82%, 91%, and 93%, respectively.

As the compliance varies with the screening tool and county, it is important to explore the influence of compliance rate to each screening tool. Therefore, a range of values between 10% and 100% were used in the sensitivity analysis to assess the impact of compliance with each screening tool.

## Results

**Figure 3:**
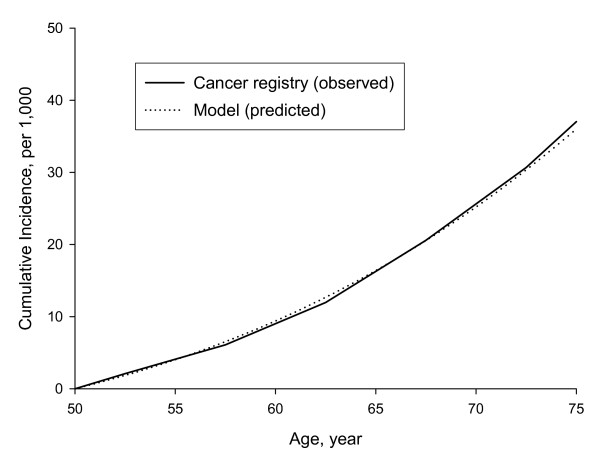
Comparison of observed and predicted cumulative colorectal cancer incidence.

The model predicted that the cumulative incidence of CRC in ages 50 to 75 years, would be 36 per 1,000, which is close to the observed cumulative incidence of CRC in Taiwan (Taiwan Cancer Registry, 37 per 1,000) (Figure 3) [17].

### Base-case analysis

Table 2 shows the simulated number of total cases of CRC, predicted number of CRC deaths, and perforation related deaths, in a cohort of 100,000 persons sub-classified by screening strategy, given 60% of compliance rate with FOBT_1_, DNA_3_, DNA_5_, and DNA_10_, 40% of compliance rate with SIGM_5 _and COLO_10_, and 85% of referral rate after a positive screening results. COLO_10 _and FOBT_1 _have similar effect on CRC mortality with 39% reduction which is the greatest one among all the screening strategies. DNA_10 _is the least effective screening strategy. The reductions in incidence with screening, compared to no screening, were very similar. However, stool DNA testing and FOBT reduces CRC incidence by far less than CRC mortality because of its poor sensitivity for adenoma.

The ICERs for other screening strategies, as compared to "No Screening" are also listed in Table 2. In this setting, COLO_10 _and FOBT_1 _are both the most cost-effective strategies which are more effective and less costly than No Screening. The incremental costs for DNA_3_, DNA_5_, and DNA_10_, respectively, were calculated as $9,794, $9,335, and $7,717 per life-year saved, which is much less cost-effective than other screening strategies. Stool DNA testing strategies were out-performed by all the other screening strategies. However, all screening strategies (including stool DNA testing) are cost-effective compared to No Screening.

### Sensitivity analysis

For identifying the influential parameters on ICER for stool DNA testing compared with No Screening, a series of sensitivity analyses were carried out (see Table 3). When the cost per stool DNA test is larger than $57.1, referral rate with diagnostic colonoscopy is lower than 67%, and prevalence of large adenoma at age 50 years is smaller than 2.42%, DNA_3 _was not cost-effective compared to $13,000 per life-year saved. Similar ICER estimates were obtained for comparisons between DNA_5_, DNA_10_, and No Screening. Besides, the discount rate also has great influence on the ICER of DNA_3_, DNA_5_, and DNA_10 _compared with No Screening. When the discount rate increases, the ICER of DNA_3_, DNA_5_, and DNA_10 _increase rapidly.

**Figure 4:**
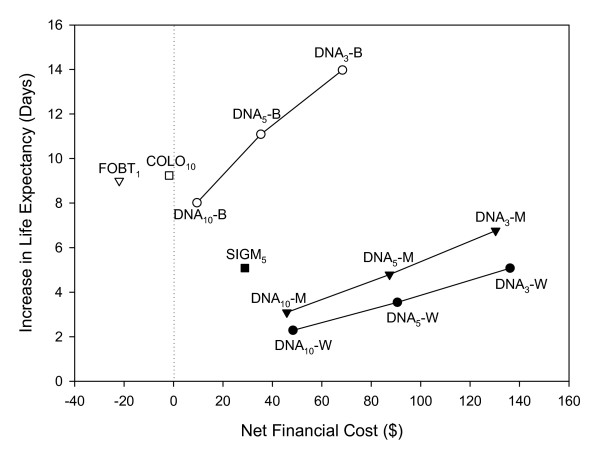
Cost-effectiveness of CRC screening under different scenarios. Abbreviations: CRC, colorectal cancer; DNA_3_, DNA_5_, and DNA_10_, stool DNA testing every 3, 5, and 10 years, respectively; -W, under the worst case scenario (the same with the base-case estimates) in which the sensitivity of stool DNA testing for small adenoma, large adenoma, and colorectal cancer, and the specificity are 8%, 15%, 52%, and 94% based on Imperiale et al study [10]; -M, under the moderate case scenario in which the corresponding estimates are 8%, 18%, 85%, and 94% based on meta-analysis [10-13, 16]; -B, under the best case scenario in which the corresponding estimates are 8%, 82%, 91%, and 93% based on Ahlquist et al study [10]; FOBT_1_, fecal occult blood testing every year; SIGM_5_, sigmoidoscopy every 5 years; COLO_10_, colonoscopy every 10 years.

Since the estimates for sensitivity of stool DNA testing vary from study to study, scenario analyses were conducted to assess the impact of sensitivity of stool DNA testing strategy (see Figure 4). In the moderate case scenario in which the sensitivities and specificity were mainly derived from the meta-analysis based on previous studies [10-13,16], the estimated ICER were similar to the base-case estimates (worst case scenario) which based on Imperiale study [16] (see two bottom curves in Figure 4). The ICER for DNA_3_, DNA_5_, and DNA_10 _are much less cost-effective than other screening strategies but still remained cost-effective compared to No Screening. Assuming the best case scenario in which the sensitivities were mainly based on the estimates from Ahlquist study [10], stool DNA testing strategies became comparable with other screening strategies, and the incremental costs for DNA_3_, DNA_5_, and DNA_10_, respectively, were calculated as $3,825, $3,036, and $2,194 per life-year saved. DNA_3 _and DNA_5 _saved more lives than FOBT_1 _and COLO_10_, but with high cost.

**Figure 5:**
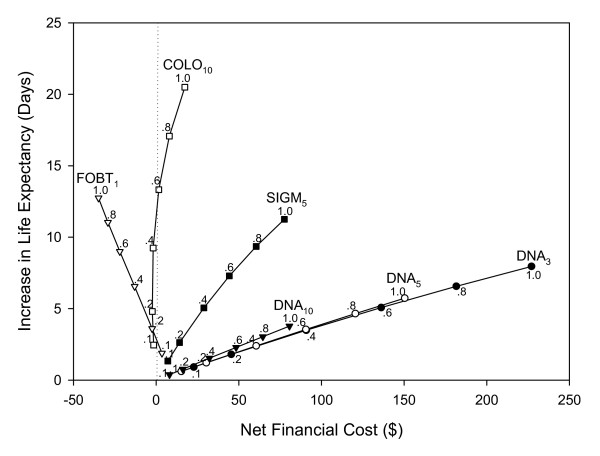
Sensitivity analysis regarding compliance to screening tool (0.1, 0.2, 0.4, 0.6, 0.8, and 1.0). Abbreviations: DNA_3_, stool DNA testing every 3 years; DNA_5_, stool DNA testing every 5 years; DNA_10_, stool DNA testing every 10 years; FOBT_1_, fecal occult blood testing every year; SIGM_5_, sigmoidoscopy every 5 years; COLO_10_, colonoscopy every 10 years.

The results of sensitivity analysis regarding compliance rate to each screening tool are illustrated in Figure 5. The effectiveness of 100% compliance to FOBT_1 _is approximately equivalent to COLO_10 _with 60% compliance rate. SIGM_5 _with 80% compliance is as effective as FOBT_1 _with 60% compliance and COLO_10 _with 40% compliance but with higher cost. DNA_3 _with 100% compliance has approximately equivalent effectiveness compared with FOBT_1 _with 50% compliance and COLO_10 _with 30% compliance but with much higher cost. The corresponding figures were FOBT_1 _with 30% compliance and COLO_10 _with 20% compliance for DNA_5_, and were FOBT_1 _with 20% compliance and COLO_10 _with 15% compliance for DNA_10_.

## Discussion

The present study is a formal economic evaluation of DNA_3_, DNA_5_, and DNA_10 _in relation to several alternatives (No Screening, FOBT_1_, SIGM_5_, and COLO_10_) using Taiwanese data on colorectal cancer incidence and disease natural history, sub-classified by adenoma size and Dukes' stage in invasive carcinoma. The findings from this study suggest that all of the screening strategies are reasonably cost-effective in relation to No Screening. However, the stool DNA testing strategies were the least cost-effective. COLO_10 _and FOBT_1 _are the most cost-effective strategy. Nevertheless, the feasibility of opting for colonoscopy as screening tool for average-risk groups may be questionable due to the shortage of medical endoscopic manpower and the risk of perforation associated with colonoscopy. Stool DNA testing would be preferable to the other screening strategies if the sensitivity for both adenoma and CRC were high enough, as seen in the best case scenario of sensitivity analysis from the Ahlquist study [10], and if the cost of the test could be lowered through economies of scale like the example of hepatitis B vaccination the price of which has dramatically fallen due to the advent of universal program in 1990s [43]. However, the interpretation of the finding in the best case scenario should be taken with great caution because subjects enrolled in Ahlquist study [10] was based on clinical series patients rather than asymptomatic subjects from average-risk population [16].

It should be noted that our focus on this study was to assess whether stool DNA testing was cost-effective provided stool DNA testing can be used as another alternative choice for population-based screening. The incremental cost-effectiveness analysis by pairwise comparison across different screening tools (i.e. colonoscopy vs. stool DNA testing) was therefore not attempted.

Although stool DNA testing has been proposed in recent years its application to mass screening for colorectal cancer has not been evaluated. The major deterrent has been doubt over the performance of test. Previous studies show a wide range of sensitivities for stool DNA testing, ranging from 15% to 82% for large adenoma and from 50% to 100% for invasive CRC [10-13,16]. This variation may often be attributed to differences in the way asymptomatic subjects or symptomatic cases were selected, although this was not the case in the Imperiale et al study [16]. The largest study to focus on asymptomatic subjects (the major target of mass screening) showed 8% sensitivity for small adenoma, 15% for large adenoma and 52% for invasive CRC [16]. False negative cases, as a result of low sensitivity, may lower its effectiveness as a tool for screening asymptomatic subjects in the general population. However, to integrate state-of-world information obtained form previous studies, we used the meta-analysis to obtain the estimate by taking all previous studies into account as the moderate case scenario. However, the estimate of sensitivity of adenoma has been largely affected by the Imperial study [16] because it is the largest study up to date on screening for colorectal cancer with stool DNA testing. The estimate based on meta-analysis for the sensitivity of cancer has been estimated as 85% by weighting the inverse variance of each study.

It could be argued that stool DNA testing screening is to be more expensive than other screening tools. This may be true at the inception of screening strategy but with widespread use, we might reasonably expect savings due to economies of scale. Genetic epidemiology is still in its infancy but the development of genetic chip technology and DNA testing advances may soon facilitate the development of simple commercial stool DNA testing kits, which would be considerably cheaper.

Very few studies have addressed the economic aspects of adopting stool DNA testing as screening tool for use in the general population. Only Song et al [44] and Leshno et al [45] have performed a study on cost-effectiveness, comparing fecal DNA testing with conventional CRC screening. The parameters in the former study are based on Western countries with a high prevalence of CRC, and the parameters in the latter study are based on Israel which also has a high incidence rate of CRC [46]. To the best of our knowledge, no similar studies on stool DNA testing have been conducted in countries with low or intermediate incidence of CRC. Furthermore, the base-case estimates of sensitivity and specificity for stool DNA testing may vary across studies. The sensitivity and the specificity used in Leshno study are 70%, 82%, 91%, and 90% for small adenoma, large adenoma, preclinical CRC, and specificity, respectively, which were similar to our best case scenario, and only annual stool DNA testing has been considered [45]. The corresponding estimates in Song study are 40%, 40%, 65%, and 95%, respectively [44]. Compared with the two previous studies [44,45], the base-case estimates in our study are the most conservative ones which only base on the population-based study [16]. However, despite the diversified parameter regarding sensitivity and specificity, our findings that stool DNA testing is cost-effective compared to No Screening but inferior to conventional screening methods such as FOBT and colonoscopy are consistent with Song et al's and Leshno et al's findings [44,45].

The reported ICERs in this study are much lower than that reported in other cost-effectiveness analysis [44,47,48]. This discrepancy can be explained by the low cost of screening tool, and other relevant cost for CRC treatment and diagnosis used in this study which represents the reality in Taiwan. When we used the cost based on the Western country as our base-case parameters [44], given perfect compliance and perfect referral rate as assumed in the previous studies, the ICERs are comparable with the previous studies [44,47,48]. The ICER for FOBT_1_, SIGM_5_, and COLO_10 _compared with No Screening are estimated as $2,376, $20,206 and $13,831 per life-year saved, respectively. However, all the stool DNA testing strategies are not cost-effective and the ICERs inflate to approximately $115,000 per life-year saved for DNA_10 _compared with No Screening.

The compliance may have large influence on the cost and the effectiveness of a screening program. Nevertheless, the compliance may vary widely by different screening tool and population [49], therefore, the decision should be made by the preference of each population. Figure 5 has shown the influence of different compliance level of each screening tool on the cost-effectiveness which allows the assessment for various compliance rates to each screening tool. For example, for a population with the compliance of 40% with FOBT_1 _and of 20% with SIGM_5 _and COLO_10_, FOBT_1_would save more lives and less costly than all the other screening strategies. However, for a population with the compliance of 50% with FOBT_1 _and of 40% with SIGM_5 _and COLO_10_, COLO_10 _would have more life-year gained than FOBT_1 _but with higher cost.

As far as the validity of our simulation model is concerned, our results obviously depend on the parameters chosen for the natural history part of the model. Three findings lead us to believe that our simulated model is adequate. Firstly, the predicted cumulative incidence of CRC in our study, 34 per 1,000, is close to the observed one, 37 per 1,000 [17]. Secondly, our sensitivity analyses demonstrates that changes to the upper and lower bounds of our estimates do not lead to substantial changes in our results. Thirdly, to check whether our simulated results on the effectiveness of FOBT test are consistent with those reported in several randomized trials [1-4], we applied 60% (56.5%~67%) compliance rate of FOBT and 85% referral rate to diagnostic colonoscopy and identical transition parameters based on base-case parameters, and shortened follow-up year to 10 years as seen in randomized trial, the predicted mortality reduction for annual FOBT screening and biennial FOBT screening is 20% and 13%, respectively, the latter of which is close to the findings from several randomised trials with two-yearly inter-screening interval, indicating 16%, 21%, and 15% mortality reduction conducted in Burgundy (France) [4], Funen (Denmark) [3], and Nottingham (UK) [2], respectively. For the incidence, the incidence ratio was estimated as 0.80 (annual FOBT) after follow-up for 18 years, which was close to the estimate reported in randomized trial [1].

Since this study was conducted from a third-party payer perspective, we therefore adopted the price mandated by the Bureau of National Health Insurance (95% population covered) as the cost and only direct costs were included. However, indirect costs such as production loss due to attending to screening or due to disease should be considered from the societal viewpoint. This could be the subject of future studies.

Our study has, however, one limitation as our proposed model assumes an adenoma to carcinoma sequence, which accounts for the majority of CRCs. Non-polypoid cases are not taken into account. However, as only a fraction of CRCs are non-polypoid and the cost-effectiveness analysis addresses the relative comparisons across screening strategies, the incorporation of the occurrence of non-polypoid tissue is unlikely to substantially affect the results.

## Conclusion

In conclusion, an economic evaluation of CRC screening with stool DNA testing was performed in a country with an intermediate incidence of CRC. Our results suggest that stool DNA testing is less cost-effective than other currently recommended strategies for population-based average-risk subjects.

## Competing interests

The author(s) declare that they have no competing interests.

## Authors' contributions

GHMW is responsible for the study design, statistical modeling, data interpretation and drafting of the article. YMW is responsible for collection and assembly of data. AMFY is responsible for statistical expertise. JMW and HCL both make great contribution to critical revision of the article for important intellectual content. JW assisted in interpreting the data and revising the article. THHC makes great contribution to conception and design, critical revision of the article for important intellectual content, statistical expertise, interpreting results, and revising manuscript.

## Pre-publication history

The pre-publication history for this paper can be accessed here:



## Figures and Tables

**Table 1:** Base-case estimates and ranges used in sensitivity analysis

Variable	Base-Case Values (Range)	Reference
The disease natural history and prognosis		

Prevalence of colorectal neoplasm at age 50 years		
Small adenoma	9.28% (4.64%–18.56%)	2–4, 17, 21*
Large adenoma	3.82% (0.96%–7.64%)	2–4, 17, 21*
Preclinical early CRC	0.12% (0.06%–0.24%)	2–4, 17, 21*
Preclinical late CRC	0.08% (0.04%–0.16%)	2–4, 17, 21*

Annual transition rates		
Normal to small adenoma (*λ*_1_(t))		
at age 50 years	0.00836 (0.00418–0.01672)	2–4, 17, 21*
at age 55 years	0.00990 (0.00495–0.01980)	2–4, 17, 21*
at age 60 years	0.01156 (0.00578–0.02312)	2–4, 17, 21*
at age 65 years	0.01333 (0.00667–0.02666)	2–4, 17, 21*
at age 70 years	0.01521 (0.00761–0.03042)	2–4, 17, 21*
Small adenoma to large adenoma (*λ*_2_)	0.0346 (0.0173–0.0692)	2–4, 17, 21*
Large adenoma to preclinical early CRC (*λ*_3_)	0.0215 (0.0108–0.0430)	2–4, 17, 21*
Preclinical early CRC to preclinical late CRC (*λ*_4_)	0.3697 (0.2678–0.4715)	22
Preclinical early CRC to clinical early CRC (*λ*_5_)	0.2382 (0.2055–0.2709)	22
Preclinical late CRC to clinical late CRC (*λ*_6_)	0.4852 (0.3207–0.6498)	22
Early CRC to CRC death (*λ*_7_)	0.0302 (0.0151–0.0604)	2–4, 23
Late CRC to CRC death (*λ*_8_)	0.2099 (0.1050–0.4198)	2–4, 23

Screening and diagnostic test characteristics		

Flexible sigmoidoscopy reach	50 (50–60)	25

Sensitivity, %		
Stool DNA testing		
Small adenoma	8 (5–75)	16
Large adenoma	15 (12–82)	10–12, 16
Preclinical CRC	52 (50–100)	10–12, 16

FOBT		
Small adenoma	5 (2–10)	26–31
Large adenoma	10 (5–20)	26–31
Preclinical CRC	50 (13–90)	26–31

Flexible sigmoidoscopy and colonoscopy		
Small adenoma	78.5 (73–84)	25
Large adenoma	85 (85–90)	25
Preclinical CRC	95 (90–100)	25, 32–38

Specificity, %		

Stool DNA testing	94 (89–100)	10–12, 16
FOBT	97.5 (95–100)	2–4
Flexible sigmoidoscopy	94 (92–96)	25
Colonoscopy	98 (96–100)	25

Complication of colonoscopy		

Perforation rate, %	0.2 (0.07–0.85)	39–40
Death rate due to perforation, per 100,000	10 (0–50)	39–40

Costs, $		

Screening		
Stool DNA testing	44.1 (22.1–88.2)	Expert opinion
FOBT	0.6 (0.3–1.2)	BNHI
Flexible sigmoidoscopy	35.3 (17.6–70.6)	BNHI
Colonoscopy	66.2 (33.1–161.8)	BNHI

Treatment and confirmation		
Pathological examination	20.6 (10.3–41.2)	BNHI
Biopsy	13.2 (6.6–26.5)	BNHI
Colonoscopy	66.2 (33.1–161.8)	BNHI
Polypectomy	42.4 (21.2–84.7)	BNHI
Initial cost for early CRC	3117.6 (1558.8–6235.3)	BNHI
Initial cost for late CRC	7705.9 (3852.9–15411.8)	BNHI
Continuing cost for CRC	176.5 (88.2–352.9)	BNHI
Terminal Care for CRC	7647.1 (3823.5–15294.1)	BNHI
Complication cost for perforation	1617.6 (808.8–3235.3)	BNHI
Complication cost for death	2735.3 (1367.6–5470.6)	BNHI

Discount rate, %	3 (0–7)	

Abbreviations: CRC, colorectal cancer; FOBT, fecal occult blood testing; BNHI, Bureau of National Health Insurance.

* The estimates were obtained and re-estimated by combining several data sources [2–4, 17, 21].

**Table 2:** Simulated results for screening strategies to prevent CRC*

Variable	Screening Strategy						
	No Screening	DNA_3_	DNA_5_	DNA_10_	FOBT_1_	SIGM_5_	COLO_10_

Total cases of CRC, n	2,917	2,435	2,654	2,710	2,129	2,253	1,780
CRC deaths, n	1,729	1,345	1,467	1,574	1,059	1,328	1,077
Perforation deaths, n	0	3	2	1	5	3	12
Reduction in CRC incidence, %	0	17	9	7	27	23	39
Reduction in CRC mortality, %	0	22	15	9	39	23	39
Life expectancy, year	15.7337	15.7476	15.7434	15.7400	15.7584	15.7477	15.7590
Total costs, thousand $	22,022	35,637	31,077	26,856	19,824	24,909	21,843
Incremental life-year saved, year	0	1,390	970	626	2,464	1,383	2,530
Incremental cost, thousand $	0	13,615	9,054	4,834	-2,198	2,887	-180
Incremental cost ($)/life-years saved compared with no screening	0	9,794	9,335	7,717	Dominant‡	2,087	Dominant‡
Incremental cost ($)/life-years saved:							
DNA_3_vs. other screening strategies	--	--	--	--	Dominant†	Dominant†	Dominant†
DNA_5 _vs. other screening strategies	--	--	--	--	Dominant†	Dominant†	Dominant†
DNA_10 _vs. other screening strategies	--	--	--	--	Dominant†	Dominant†	Dominant†

Abbreviations: CRC, colorectal cancer; DNA_3_, stool DNA testing every 3 years; DNA_5_, stool DNA testing every 5 years; DNA_10_, stool DNA testing every 10 years; FOBT_1_, fecal occult blood testing every two year; SIGM_5_, sigmoidoscopy every 5 years; COLO_10_, colonoscopy every 10 years.

* Values obtain from a cohort of 100,000 persons 50 years of age who were followed for 25 years.

† The other screening strategy is more effective and less costly than stool DNA testing strategy.

‡ The screening is more effective and less costly than No Screening.

**Table 3:** One-way sensitivity analysis.

Variables	Base- Case Values	Sensitivity Analysis Values	Incremental Cost($)/Life-Year Saved					
			
			DNA_3_		DNA_5_		DNA_10_	
			
			Range	Threshold	Range	Threshold	Range	Threshold
Base-case			9,794		9,335		7,717	
Sensitivity of DNA for small/large adenoma	8%/15%	75%/82%	11,546 – 1,742	--	11,122 – 1,030	--	9,369 – 269	--
Sensitivity of DNA for preclinical CRC	52%	50%–100%	9,921 – 7,078	--	9,454 – 6,752	--	7,817 – 5,528	--
Cost per DNA	$44.1	$22.1-$88.1	4,344 – 2,0706	$57.1	4,194 – 19,628	$60.0	3,271 – 1,6618	$70.3
Compliance with DNA	60%	10%–100%	9,070 – 10,423	--	9,047 – 9,582	--	7,630 – 7,787	--
Referral rate to diagnostic colonoscopy	85%	10%–100%	85,266 – 8,294	67%	83,868 – 7,843	63%	73,826 – 6,396	53%
Prevalence of large adenoma	3.82%	0.96%–7.64%	18,969 – 5,387	2.42%	18,184 – 5,114	2.23%	15,657 – 4,042	1.58%
Discount rate	3%	0%–7%	6,938 – 15,674	--	6,738 – 14,864	--	5,107 – 13,281	--

DNA_3_, stool DNA testing every 3 years; DNA_5_, stool DNA testing every 5 years; DNA_10_, stool DNA testing every 10 years; FOBT_1_, fecal occult blood testing every two year; SIGM_5_, sigmoidoscopy every 5 years; COLO_10_, colonoscopy every 10 years.
